# Innovative Educational Tools Facilitating Lifestyle and Nutritional Interventions in Type 1 Diabetes Mellitus

**DOI:** 10.3390/nu17061026

**Published:** 2025-03-14

**Authors:** Christos Daramilas, Maria Papagianni, Christina Ntafi, George Mastorakos, Chrysanthi Sardeli

**Affiliations:** 1 Laboratory of Clinical Pharmacology, School of Medicine, Faculty of Health Sciences, Aristotle University of Thessaloniki, 54124 Thessaloniki, Greece; chridara@auth.gr (C.D.); cntafi@hotmail.com (C.N.); 2 Department of Nutrition and Dietetics, School of Physical Education, Sport Science and Dietetics, University of Thessaly, 42132 Trikala, Greece; mpapag@uth.gr; 3 3rd Department of Pediatrics, School of Medicine, Faculty of Health Sciences, Aristotle University of Thessaloniki, Ippokrateio Hospital of Thessaloniki, 54642 Thessaloniki, Greece; 4 Unit of Endocrinology, Diabetes Mellitus and Metabolism, Aretaieion Hospital, Athens Medical School, National and Kapodistrian University of Athens, 11527 Athens, Greece; mastorakg@gmail.com

**Keywords:** type 1 diabetes mellitus, educational tools, educational guide, educational maps, lifestyle, nutrition, patient education

## Abstract

**Background/Objectives:** Education plays a crucial role in encouraging and aiding diabetic patients to take active responsibility for the day-to-day management of their condition and can reduce disease burden, thus minimizing the risk of complications, as well as morbidity and mortality. Lack of information increases the chances of diabetes complications. **Methods:** A combination of conceptual allegory and group presentation of experiences using appropriate, tailor-made, educational material was applied. To better support the trainees, maps for diabetes education were created, an educational tool that gathers roles and procedures, guiding diabetic patients in their self-care and supporting them in being able to develop coping mechanisms and self-manage their disease. The educational material was used during educational sessions held in three Greek cities. The effect of patient education was measured using specifically developed questionnaires at two time points, namely, before and after the implementation of the intervention. **Results:** The results showed that training using innovative educational tools had a positive effect on the lifestyle and nutrition of diabetic patients, as their health indicators improved, (significant decreases in HbA1c, incidents of severe hypoglycemia, and emotional distress, as well as improvements in self-reported hypoglycemia awareness and wellbeing were observed), without changes in the prescribed pharmacotherapy. **Conclusions:** The need for structured educational courses on self-management for individuals with T1DM is indisputable. The main objective of such a program should be the motivation of patients to be actively involved in the prevention and management of both diabetes and its complications.

## 1. Introduction

Diabetes and its complications pose a sizable public health burden, as diabetes mellitus (DM) is currently one of the most common chronic diseases.

Although the treatment of diabetes in recent years has changed drastically due to scientific innovations in internal medicine and pharmacology, adequate diabetes control remains difficult to maintain in clinical practice, and several studies have documented that suboptimal control of plasma glucose, plasma lipids, and blood pressure remains common in diabetic patients [1]. Moreover, technological innovations have had a positive impact on diabetes’ outcomes, including improvements in hemoglobin A1C (HbA1c) levels, and diabetes self-management behaviors and can benefit people living with diabetes when used in conjunction with care delivered by healthcare professionals [2].

The American Diabetes Association defines diabetes self-management education as the ongoing process of facilitating knowledge, skills, and abilities necessary for diabetes self-care, that incorporates a person-centered approach and shared decision making [3]. In the UK, NICE also endorses diabetes’ self-management education and states that it should consist of an evidence-based, structured curriculum, with specific aims and objectives delivered by trained educators and outcomes that are audited regularly against consistent criteria by independent assessors for quality assurance [4,5].

In this regard, the optimal management of DM includes not only adequate glycemic control but also preventing and treating diabetic complications, both micro- and macrovascular, as well as improvement of overall quality of life. Active patient involvement and self-care behaviors, including adequate blood glucose monitoring, healthy eating, physical activity, developing robust problem-solving and coping skills, compliance with pharmacotherapy, and adopting risk reduction behaviors, have been correlated with improved glycemic control, body fat reduction, fewer diabetic complications, and improved quality of life in diabetic patients [6].

The serious and chronic nature of diabetes, the intricacy of its management, and the numerous daily self-care decisions that living with diabetes requires mean that adherence to a predetermined care program is not easily achieved [7]. This is especially true when the self-management plan has been designed to tackle the disease but has not been tailored to fit the patients’ priorities, goals, resources, culture, and lifestyle. For effective diabetes management, patients must be able to set goals and make daily decisions that are both effective and fitting their values and routines, while taking into account numerous subjective physiological and psychosocial factors [8].

An Australian systematic review of thirty-two studies found that most people with diabetes self-manage, although there is variation in adherence to key self-management activities, with adherence to pharmacotherapy being highest of all, whereas substantial variation exists under special circumstances for self-monitoring of blood glucose, nutritional changes, motion, and foot care [9].

Educational and psychosocial interventions can improve diabetes management [10]. A systematic review by Hampson et al. [10] investigated the educational and psychosocial intervention efficacy on improvement of diabetes management in adolescent T1DM patients. It showed that educational and psychosocial interventions have positive effects on several diabetes management consequences.

Structured educational programs have proven to be valuable in ensuring that patients with DM are provided with the required knowledge and skills to modify their behavior and to better self-manage their disease and its complications. In this context, various educational programs are available for diabetic patients, including the DAFNE (Dose Adjustment For Normal Eating) program, a 5-day course with a focus on carbohydrate intake estimation, insulin dose adjustments, and glucose self-monitoring. The DAFNE program was shown to improve HbA1c levels and quality of life in T1DM patients (n = 169) in a randomized controlled trial [11]. More recently, the implementation of the DAFNE course in a routine clinical setting of T1DM patients (n = 639) in the UK led to significant improvement in HbA1c levels, reduction in episodes of severe hypoglycemia, and psychological distress, as well as improvement in self-reported hypoglycemia awareness and wellbeing 1 year post-intervention [12]. In Australia, DAFNE achieved similar significant HbA1c improvement at 1 year, with a mean HbA1c reduction of 0.4%, weight reduction (0.9 kg), reduced frequency of severe hypoglycemia, and improvement in quality of life [13]. Further improvements following the Australian DAFNE program included a reduction in severe diabetes-related distress (29.3% [n = 145] vs. 12.6% [n = 60], *p* < 0.001) and episodes of diabetic ketoacidosis (4.1% [n = 20] to 1.2% [n = 6]) [14]. Programs similar to DAFNE have shown similar findings in Austria [15], Germany [16,17], Switzerland [18], and France [19]. Comparable outcomes have been obtained by other studies. There is, however, a noteworthy lack of robust evidence to support this practice, especially in adult patients. Finally, the effect of such interventions of longer duration (1 year) has not been assessed. To achieve optimal management of DM, the main conditions are the following: (i) taking appropriate medications; (ii) education in self-management of the condition [20]. According to a systematic review [20] on diabetes awareness and the effectiveness of diabetes education, a diabetes education program should include the following measures: (i) encouraging and supporting patients to play an active role in the daily management of their condition; (ii) providing detailed information to reduce the chances of developing complications of DM; (iii) providing information related to the risk of severe hypoglycemia increased by reduced awareness of hypoglycemia; (iv) improving education and knowledge about self-monitoring and pharmacotherapy at the right time, so to decrease risk factors as well as the chances of complications, thereby reducing morbidity and mortality in diabetics.

There are two educational approaches that the global scientific community utilizes to educate patients with diabetes. The first is traditional DM education that focuses on self-management and educational resources about DM across its spectrum, again through group sessions. The second involves empowerment through group sessions that incorporate two educational innovations, i.e., conceptual allegory to promote insight and group methods of presenting experiences to promote active learning [21].

The present study focuses on examining whether a six-month-long educational program with tools that motivates T1DM patients to follow a Mediterranean diet, as well as regulate carbohydrate intake and insulin dose as indicated and perform exercise regularly, could result in improved metabolic control among adult patients and parents on behalf of minors compared to the results obtained for the same adults and parents on behalf of minors before they joined this educational program with tools.

## 2. Materials and Methods

One hundred and five (105) patients with T1DM or parents of minors with T1DM, who consented to participate in the educational program after receiving appropriate information, were recruited from the members of 3 local chapters of the Panhellenic Federation of Associations of People with Diabetes in Greece (P.O.S.S.A.S.DIA.), residing in three cities of Peloponnese, Greece, namely, Sparti, Kalamata, and Tripoli. Prior to participation, all participants received the necessary oral and written information, as well as preapproved consent and withdrawal of consent forms and signed to indicate acceptance of participation.

The education group (EG) consisted of 61% females and 39% males, aged 10 to 76 years, with parents participating on behalf of 14 minors. The mean EG BMI was 26.2 ± 4.9 kg/m^2^; for the adults, the BMI was 26.9 ± 4.4 kg/m^2^, and for the minors, 20.2 ± 10.9 kg/m^2^. Employing a pretest–posttest study design, data from the same patients before their participation in the educational program were used.

The EG consisted of seventy-three patients on intensive insulin therapy (n = 73) and thirty-two patients on continuous insulin pump infusion (n = 32). The diabetes treatment plan remained the same throughout the intervention, except for slight adjustments in insulin dosing (up to 20% of the total insulin dose), as indicated. Seventy-four patients without complications participated in the educational program, along with twenty with ophthalmological complications, eight with cardiovascular complications, one with nephrological complications, and one with neurological complications. All participants completed the 6-month-long course.

The educational method applied was a combination of two methodologies, namely, conceptual allegory and group presentation of experiences, using appropriate tailor-made educational material. To better support the trainees, maps for education in type 1 diabetes were created. These maps are a series of pictorial guides via which participants are involved in conversation, sharing beliefs and experiences about their lives with diabetes. A healthcare professional functions as a facilitator, steering the conversation. The participants acquire knowledge and data about diabetes self-management and care. The maps present recognizable situations, through which diabetic persons can easily comprehend different facets of diabetes that correlate to their personal experiences [22].

The maps employ images, questions, data, and scientific information to provide an interactive and participatory learning experience for people with diabetes. These maps are intended to provide a framework for dynamic, supportive, and group discussion and contribute to a learning process that inspires the participants to take charge of their own health care, always in collaboration and as prescribed by their healthcare practitioners.

The intervention lasted 6 months, with 8 h sessions held for two consecutive days, every month. Each educational session was 270 min long. The participants were encouraged to adopt a Mediterranean diet and workout regularly and to adjust their carbohydrate intake and insulin dose as indicated. The training program included 27 educational modules, on which participants were trained. At the beginning and at the end of the program, all the participants were asked to fill in specifically designed questionnaires. When starting each session, a lengthy conversation was held regarding the application of knowledge gained during the previous day-sessions and its efficacy in problem-solving. The sessions took place in two big groups, each group consisting of the physician in charge (an internist specialized in diabetes care), a diabetes educator (a healthcare professional, himself a T1DM patient), and 6–8 T1DM patients. All participants completed all modules—either on the initially planned dates or via participation in catch-up sessions.

Three different visual maps were used. Each map is a 3-by-5-foot tabletop board depicting drawings of situations known to diabetic patients. In each session, a map was placed on a board, with the participants seated in a U formation around it. The first map, titled “What is type 1 diabetes mellitus”, provides an overview of the mechanisms that regulate insulin secretion in the pancreas and the role of the pancreas and insulin in modulating blood glucose levels and is designed to explain how diabetes happens and how insulin therapy and emotions could be managed. The second map, named “Daily life with type 1 diabetes mellitus”, provides information about the handling of high and low blood glucose levels, the importance of self-monitoring, diet, and exercise, and the proper use of new technologies. The third map, titled “Healthy eating and exercise”, focuses on healthy nutrition and exercise, discusses terms like glycemic index, and provides key features of healthy eating.

As with the contemporary approach, the sessions were culturally mindful and adapted to the participants’ habits [23]. The participants were allowed to observe their usual eating routines while participating in the study. However, they were encouraged to adopt a Mediterranean diet and were told to calculate the amount of carbohydrates they were consuming and to alter their insulin dose as indicated. The patients were also prompted to exercise regularly, for 1 hour at least thrice weekly, at a moderate pace. Guidance was offered as to carbohydrate consumption and indicated insulin dose modifications.

The primary outcomes included changes in HbA1C levels and early detection of hypoglycemic incidents at the beginning and end of the intervention. Furthermore, a record was kept of body weight at the same timepoints. Descriptive statistics are presented in terms of numbers, means, standard deviations, percentages, and ranges. Data management and statistical analysis were accomplished using Microsoft Excel, including additional tools (Microsoft Office XP Professional). The study was conducted in accordance with the principles outlined in the Declaration of Helsinki and approved by the Ethics Committee of the School of Medicine of the Aristotle University of Thessaloniki, Greece (Approval Decision Nr. 240/2-7-2024).

## 3. Results

The HbA1c values were significantly reduced after the six-month-long intervention (7.7 ± 1.8% vs. 6.7 ± 1.1%, *p* < 0.05). The reduction in the HbA1c levels was significant in both adults (7.7 ± 2% vs. 7 ± 0.9%) and minors (7.7 ± 1% vs. 6.6 ± 0.3%). At the end of the study period, the HbA1c levels were significantly reduced in both sexes and in all age groups, followed by a reduction in hypoglycemia, compared to the data before the implementation of the educational program. The mean body weight did not change, but there was a decrease in the range of weight variation in the whole group. Relevant results of the intervention are depicted in Table 1.

The questionnaires filled out by the participants at baseline and at the end of the intervention period were divided into four thematic sections:

1. Diabetes-related issues that may currently be a problem for people with diabetes who participated (five questions);
2. Questions related to their perception regarding taking insulin to manage their diabetes (nine questions);
3. Behavioral changes in daily routine to prevent hypoglycemia and its consequences (eight questions);
4. Concern related to not recognizing hypoglycemia (one question).

The first thematic section focused on the emotions caused by the presence of diabetes in the participants’ lives before and after the intervention. Five questions were asked, of which the three most significant ones are presented in Table 2a.

As observed, the educational intervention, which solved problems and provided answers to questions, helped to reduce the feelings of “fear” and “anxiety” about the future significantly. It is noteworthy that for the last question, related to displaying complications from diabetes in the future, despite a general decrease in the rate of worry, the percentages in the answers “somewhat serious problem” and “serious problem” were 20% and 21%, respectively, remaining at a high level, suggesting that despite the fact that the participants received overall education about diabetes, they did not feel completely secure about the future.

The second theme focused on insulin and knowledge about the management of diabetes with the use of insulin. Nine questions were asked, of which eight are presented in Table 2b. The educational intervention helped to reduce the fear of insulin therapy and its perceived risk of causing hypoglycemia.

The third theme focused on the possible behavioral changes that the participants may have had to undergo in trying to prevent hypoglycemia and its consequences. Eight questions were asked, of which five are presented in Table 2c. The intervention succeeded in improving behavioral patterns among the participants.

The fourth theme focused on undiagnosed hypoglycemia, a very serious phenomenon that can causes death. The question asked is presented in Table 2d. To the question “I didn’t feel/realize I had hypoglycemia” before the educational intervention, the responses that garnered the highest percentages were “Rarely” with 30.5% and “Sometimes” with 39%. After the educational intervention, the responses that garnered the highest percentage were “Never” with 56.2% and “Rarely” with 24.8%, while “Sometimes” decreased to 19%.

## 4. Discussion

As presented in the Introduction, the goal of diabetes self-management education (DSME) is to aid patients to take control of their condition by increasing their knowledge and improving their skills to make informed choices for self-directed behavioral change, allowing them to integrate self-management into their everyday lives and eventually to decrease the risk of complications.

One hundred and five patients (105) with T1DM participated in the present study; of those, 91 were adults, and 14 were parents of minors with T1DM. The participants completed a structured educational program for self-monitoring and diabetes management. The educational intervention presented aimed at discussing the pathophysiology and treatment of T1DM, along with preventing and managing its complications. Additionally, the intervention aimed at recognizing, preventing, and managing hypoglycemic incidents, because hypoglycemia can considerably affect not only the treatment of T1DM but also the quality of life of patients. The reduction in the HbA1C levels post-intervention is consistent with the available literature [24,25,26,27,28,29]. Abolfotouh et al. assessed the effect of four educational courses over a 4-month period in 503 adolescents with T1DM and found an improvement in quality of life and a small decrease in the HbA1c values [24]. In addition, Mannucci et al. reported a significant decrease in the HbA1c levels, from 7.5 ± 1.8 to 6.8 ± 1.4%, but only in patients who followed a similarly structured approach and were willing to participate [25]. In a related study, weekly psychoeducational sessions (n = 7) with 60 patients with T1DM also resulted in a 0.3% reduction in HbA1c in 6 months, whereas no changes were recorded in the control group [26]. Finally, in another study, in 25 uncontrolled adolescents with T1DM, a 3-month psychoeducational program (which included one session for parents and three sessions for participants) significantly reduced the HbA1c levels by 0.65% at the end of a 9-month follow-up period [27]. Nevertheless, not all educational programs succeed in enhancing glycemic control. In this context, the Child and Adolescent Structured Competencies Approach to Diabetes Education (CASCADE) program was provided to 362 patients with T1DM and their families without improving the HbA1c levels during follow-up [28]. Likewise, according to available evidence from interventions targeting African American diabetics, a decrease in HbA1C of only 0.8% is expected [29]. However, a meta-analysis did not find significant improvement achieved through behavioral programs for T1DM, but, as the authors noted, all studies included in the analysis showed a moderate to high risk of bias [30].

In the present study, compared with previous studies, the data show a greater reduction in the HbA1c values (0.71%) (Table 1 and Table 2). This difference may well be credited to the longer duration of the program, to the particular toolkit that was constructed and used in the study, or to its adaptation to local nutritional and cultural habits, an approach that improves patient adherence [22]. Indeed, it has been found that the group context, as evident in the present study, is more successful than the individual one [31]. Other factors that contributed to the marked improvement in HbA1c are the fact that this was not a randomized controlled trial but included only patients who were eager to join in. It has been previously reported that patients who wish to improve their glycemic control and who are anxious about their disease are more likely to adopt and adhere to new treatment strategies [32]. In the present study, along with the improvement in HbA1c, a major change in the participants’ perception of DM and its risks was observed. The results infer a significant reduction in fears related to the future and the complications that DM may cause if not managed. The intervention succeeded in educating the participants to properly manage their glycemic control and thus avoid the complications that the condition can cause. In addition, the intervention succeeded in reducing unrecognized hypoglycemia and the negative emotions, especially fear, that it caused [33]. Myths concerning the use of insulin were busted, and the participants were educated on its correct use and administration. In DM, according to many scientific groups, knowledge and management of food is the cornerstone in the regulation of the condition. Once the knowledge of appropriate food selection and the correct processing of food-related information is achieved, then, and only then, is euglycemia likely to be achieved. Miscalculation of food carbohydrates/lipids/proteins leads to miscalculation of insulin doses, hence a glycemic outcome that will be either hypoglycemic or hyperglycemic. In addition, the role of the glycemic index in the two-hour postprandial glucose value is a parameter that, if not correctly assessed, cannot give us the desired results in HbA1c measurement.

Overall, it seems that cultural adaptation, longer duration of each educational session, use of alternative methods of education, use of a group setting, and self-motivation of the patients contribute to increased adherence and improvement both at a psychological level and at the level of the HbA1c results, which serves as an indicator for the evaluation of all parameters such as diet, management of hypoglycemia and hyperglycemia, exercise, hormonal imbalance wherever it is caused, and others. We consider the results on this question a success because we were able to completely reverse the perception of the severity of unrecognized hypoglycemia. This finding is of great significance in daily practice, since hypoglycemia continues to be a critical issue in the management of patients with T1DM [34,35]. Furthermore, it should be stressed that being afraid of hypoglycemia may further affect negatively the glycemic control in T1DM and has also been correlated with cognitive dysfunction in diabetic minors [36,37].

Employing such an approach presents some planning and budgetary challenges; therefore, alternatives have been proposed to increase the efficacy of such training interventions, including internet-based programs [38,39,40]. Apps for smartphones and smart watches collecting data on motion, diet, insulin therapy, and self-monitoring have also been developed [41]. A recent meta-analysis confirmed the importance of such interventions combined with technology-based programs in improving hypoglycemia awareness and glycemic control in patients with T1DM [42].

The principal limitation of this study is that it was not a randomized controlled trial. This could have affected the results, since the study group consisted of patients who had chosen to participate rather than being randomly assigned to a group. Consequently, it can be assumed that the participants were more motivated than their peers to increase their glycemic control, and this factor may explain any change observed before and after the educational intervention. Conversely, since this study reflects real-world data, one expects that the active participation of patients will be reflected in their DM status, and thus, the educator’s role is to support and guide them to stick to the rules, something which can certainly be achieved by repeating the training process after two years, a reasonable period of time, to reassess their health status.

## 5. Conclusions

To conclude, this study clearly showed significant improvements in glycemic control and prevalence of hypoglycemia in T1DM patients who attended a DM educational program. These results validate the need for regularly providing structured educational courses on self-management in patients with T1DM. Data on the pathophysiology and treatment of diabetes and its complications should be incorporated in such programs to increase patients’ awareness of DM. The main objective of such a program should be to motivate the participants or their caretakers to actively participate in the daily management of the disease and its complications. The success of such an approach seems to depend on employing sessions of longer duration, a teamwork approach, a commitment to active involvement in achieving glycemic control, and the adaptation of the educational material and sessions to ethnic and cultural differences. The results of this study agree with Dr. Joslin’s famous quote stating that “The person with diabetes who knows the most lives the longest” [43].

## Figures and Tables

**Table 1:** Results of the comparison between beginning and end of the intervention period.

	Before (n = 105)	After (n = 105)
** Weight (kg) **	72.2 kg (Min = 28 kg/Max = 110 kg)	72.5 kg (Min = 32 kg/Max = 106)
** BMI (kg/m^2^) **	26.2 ± 4.9 (kg/m^2^)	26.3 ± 3 (kg/m^2^)
** HbA1c (%) (IFCC mmol/mol) **	7.7% (Min = 6.5%/Max = 10%)	6.99 % (Min = 6 %/Max = 8.2 %)
**Identification of hypoglycemia symptoms**	Never + Rarely = 42.9%	Never + Rarely = 81%
**Worry about developing hypoglycemia**	Sometimes = 39%	Sometimes = 19%

**Table 2:** (a) Diabetes-related issues that may currently be a problem for participants. (b) Questions related to participants’ perception regarding using insulin to manage their diabetes. (c) Behavioral changes in daily routine to prevent hypoglycemia and its consequences. (d) Concerns about not recognizing hypoglycemia.

(a)						
Statement		It’s Not a Problem	Small Problem	Medium Problem	Somewhat Serious Problem	Serious Problem
**Do you feel fear when you think about living with diabetes?**	Before ([%] n)	16.2% (17)	26.7% (28)	33.3% (35)	12.4% (13)	11.4% (12)
	After ([%] n)	35.2% (37)	27.6% (29)	24.8% (26)	8.6% (9)	3.8% (4)
**Do you feel depressed when you think about living with diabetes?**	Before ([%] n)	18.1% (19)	28.6% (30)	28.6% (30)	19% (20)	5.7% (6)
	After ([%] n)	36.2% (38)	31.4% (33)	17.1% (18)	12.4% (13)	2.9% (3)
**Are you worried about the future and the possibility of serious complications?**	Before ([%] n)	1.9% (2)	12.4% (13)	19% (20)	33.3% (35)	33.3% (35)
	After ([%] n)	13.3% (14)	24.8% (26)	21% (22)	20% (21)	21% (22)
**(b)**						
**Statement**		**I Totally Disagree**	**I Disagree**	**Neither Disagree nor Agree**	**Agree**	**Strongly Agree**
** Receiving larger doses of insulin means my diabetes has become worse **	Before ([%] n)	12.4% (13)	26.7% (28)	30.5% (32)	26.7% (28)	3.8% (4)
	After ([%] n)	7.6% (8)	70.5% (74)	13.3% (14)	2.9% (3)	5.7% (6)
** Receiving insulin helps to prevent complications of diabetes **	Before ([%] n)	6.7% (7)	4.8% (5)	36.2% (38)	23.8% (25)	28.6% (30)
	After ([%] n)	0% (0)	0% (0)	5.7% (6)	61% (64)	33.3% (35)
**I’m afraid to inject myself with insulin using a needle**	Before ([%] n)	33.3% (35)	24.8% (26)	29.5% (31)	10.5% (11)	1.9% (2)
	After ([%] n)	30.5% (32)	56.2% (59)	2.9% (3)	1% (1)	9.5% (10)
**Insulin therapy increases the risk of hypoglycemia**	Before ([%] n)	17.1% (18)	23.8% (25)	28.6% (30)	28.6% (30)	1.9% (2)
	After ([%] n)	4.8% (5)	7.6% (8)	13.3% (14)	53.3% (56)	21% (22)
**Insulin helps improve my health**	Before ([%] n)	6.7% (7)	3.8% (4)	34.3% (36)	31.4% (33)	23.8% (25)
	After ([%] n)	1% (1)	0% (0)	9.5% (10)	24.8% (26)	64.8% (68)
** Taking insulin causes me to increase my weight **	Before ([%] n)	10.5% (11)	23.8% (25)	43.8% (46)	20% (21)	1.9% (2)
	After ([%] n)	17.1% (18)	26.7% (28)	35.2 (37)	21% (22)	0% (0)
** Receiving insulin by injection is painful **	Before ([%] n)	17.1% (18)	28.6% (30)	41% (43)	13.3% (14)	0% (0)
	After ([%] n)	21% (22)	69.5% (73)	0% (0)	5.7% (6)	3.8% (4)
**Receiving insulin helps to keep blood glucose in range**	Before ([%] n)	3.8% (4)	4.8% (5)	30.5% (32)	36.2% (38)	24.8% (26)
	After ([%] n)	1% (1)	0% (0)	7.6% (8)	50.5% (53)	41% (43)
**(c)**						
**Statement**		**Never**	**Rarely**	**Sometimes**	**Often**	**Almost Always**
**I tried to keep my blood glucose levels above 150 mg/dl**	Before ([%] n)	13.3% (14)	22.9% (24)	37.1% (39)	21.9% (23)	4.8% (5)
	After ([%] n)	27.6% (29)	38.1% (40)	16.2% (17)	11.4% (12)	6.7% (7)
**I reduced my insulin dose when my glucose levels were low**	Before ([%] n)	8.6% (9)	15.2% (16)	41.9% (44)	26.7% (28)	7.6% (8)
	After ([%] n)	2.9% (3)	2.9% (3)	16.2% (17)	24.8% (26)	53.3% (56)
**I reduced my exercise/physical activity**	Before ([%] n)	39% (41)	25.7% (27)	19% (20)	15.2% (16)	1% (1)
	After ([%] n)	53.3% (56)	30.5% (32)	14.3% (15)	1% (1)	1% (1)
**I kept my blood glucose level higher than the desired range when doing important work**	Before ([%] n)	7.6% (8)	24.8% (26)	38.1% (40)	22.9% (24)	6.7% (7)
	After ([%] n)	12.4% (13)	38.1% (40)	30.5% (32)	19% (20)	0% (0)
** Other people checked my glucose level several times during the day or night **	Before ([%] n)	21% (22)	32.4% (34)	27.6% (29)	12.4% (13)	6.7% (7)
	After ([%] n)	39% (41)	39% (41)	7.6% (8)	5.7% (6)	8.6% (9)
**(d)**						
**Statement**		**Never**	**Rarely**	**Sometimes**	**Often**	**Almost Always**
**I didn’t feel/realize I had hypoglycemia**	Before ([%] n)	12.4% (13)	30.5% (32)	39% (41)	18.1% (19)	0% (0)
	After ([%] n)	56.2% (59)	24.8% (26)	19% (20)	0% (0)	0% (0)

## Data Availability

The original contributions presented in the study are included in the article, further inquiries can be directed to the corresponding author.
